# Rifampin phosphotransferase is an unusual antibiotic resistance kinase

**DOI:** 10.1038/ncomms11343

**Published:** 2016-04-22

**Authors:** Peter J. Stogios, Georgina Cox, Peter Spanogiannopoulos, Monica C. Pillon, Nicholas Waglechner, Tatiana Skarina, Kalinka Koteva, Alba Guarné, Alexei Savchenko, Gerard D. Wright

**Affiliations:** 1 Department of Chemical Engineering and Applied Chemistry, University of Toronto, Toronto, Ontario, Canada M5G 1L6; 2 M.G. DeGroote Institute for Infectious Disease Research, Department of Biochemistry and Biomedical Sciences, McMaster University, 1280 Main St W, Hamilton, Ontario, Canada L8S 4K1; 3 Department of Biochemistry and Biomedical Sciences, McMaster University, 1280 Main St W, Hamilton, Ontario, Canada L8S 4K1

## Abstract

Rifampin (RIF) phosphotransferase (RPH) confers antibiotic resistance by conversion of RIF and ATP, to inactive phospho-RIF, AMP and P_i_. Here we present the crystal structure of RPH from *Listeria monocytogenes* (RPH-*Lm*), which reveals that the enzyme is comprised of three domains: two substrate-binding domains (ATP-grasp and RIF-binding domains); and a smaller phosphate-carrying His swivel domain. Using solution small-angle X-ray scattering and mutagenesis, we reveal a mechanism where the swivel domain transits between the spatially distinct substrate-binding sites during catalysis. RPHs are previously uncharacterized dikinases that are widespread in environmental and pathogenic bacteria. These enzymes are members of a large unexplored group of bacterial enzymes with substrate affinities that have yet to be fully explored. Such an enzymatically complex mechanism of antibiotic resistance augments the spectrum of strategies used by bacteria to evade antimicrobial compounds.

## Abstract (web summary)

Antibiotic resistance is a major clinical problem that threatens to undermine our ability to control infectious diseases. Here the authors present detailed structural analysis of Rifampin phosphotransferase from *Listeria monocytogenes*, yielding insight on how this class of enzyme inactivates its target antibiotics.

## Graphical abstract

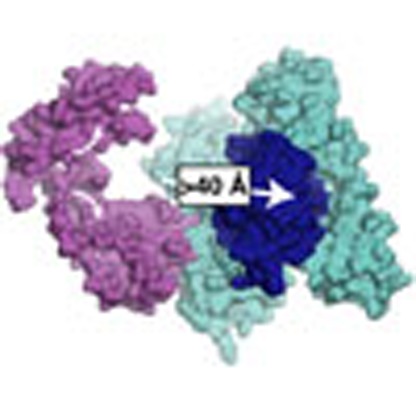

The ability of bacteria to respond and adapt to antibiotics has led to a severe reduction in the efficacy of our anti-infective drugs^1^. The origins of many antibiotic resistance elements lie within the genomes of non-pathogenic environmental bacteria that are intrinsically resistant to many classes of antibiotics^2^,^3^,^4^. This reservoir of genes predates the selective pressure imposed by the clinical application of antibiotics^5^,^6^,^7^,^8^. Furthermore, these resistance genes have evolved from, or share common ancestors with, proto-resistance genes encoding proteins that have other cellular functions^9^. Notable examples include aminoglycoside and lincosamide nucleotidyltransferases (*ant* and *lnu* genes), which exhibit ancestral connections to DNA polymerases^10^,^11^,^12^; the aminoglycoside acetyltransferase (*aac(2′)-Ia*) from *Providenica stuartii*, which is thought to modify peptidoglycan under its native conditions^13^; the chloramphenicol kinase that is closely related to both shikimate and adenylate kinases^14^; and aminoglycoside/macrolide phosphotransferases that exhibit functional and structural similarities to lipid and protein kinases^15^,^16^. In most of these cases, due to low amino acid sequence homology, structural studies have been necessary to reveal the relationship between proto- and bona fide resistance genes.

The rifamycin antibiotics, such as rifampin (RIF) (Fig. 1a), are first line treatments for mycobacterial infections, including those caused by *Mycobacterium tuberculosis*^17^. Rifamycins are also increasingly used as adjunct therapies to treat drug-resistant *Staphylococcus aureus*, and as prevention of traveller's diarrhoea and hepatic encephalopathy. In *M. tuberculosis*, resistance to this family of antibiotics is most often conferred by point mutations in the drug target, RNA polymerase^18^; however, additional rifamycin resistance mechanisms exist in other species of bacteria^19^. These include enzymatic inactivation of rifamycins by glycosylation^20^, ADP ribosylation^21^ and phosphorylation^22^. We recently identified and characterized the first example of a RIF phosphotransferase (RPH)^23^. This study revealed that *rph* orthologs are widespread in organisms phenotypically sensitive to RIF, such as the human pathogen *Listeria monocytogenes*^23^. However, these genes were shown to be capable of conferring high-level RIF resistance when heterologously expressed^23^. The existence of *rph* genes in these phenotypically susceptible organisms is an example of ‘silent resistance'^24^,^25^. Silent resistance genes only confer resistance when mobilized from their native setting. A good example of this phenomenon is chromosomally located Enterobacteriaceae β-lactamases, which confer weak resistance in their host organism because of poor expression, but confer high-level resistance when their expression levels increase due to mobilization into plasmids or acquisition of promoter mutations^24^,^26^,^27^.

A search for RPH homologues revealed the presence of members of this family in many bacterial genomes, often misannotated as phosphoenolpyruvate synthases (PEPS) and pyruvate phosphate dikinase (PPDK). PEPS and PPDK enzymes catalyse the conversion of pyruvate and ATP to PEP, AMP and P_i_ in a two-step process^28^,^29^,^30^,^31^. They are composed of three domains, an N-terminal ATP-grasp domain, a C-terminal domain that binds pyruvate, and a small swivel phosphohistidine domain that harbours the conserved histidine residue essential for catalysis. RPH similarity with PEPS and PPDK is restricted to the ATP-grasp and swivel phosphohistidine domains, while the rest of the RPH sequence exhibits relatively low amino acid similarity with these enzyme families. Furthermore, the domain architecture of RPH differs in that the catalytic histidine (His_825_) responsible for phosphorylation of RIF is found at the C-terminal region of the protein rather than at the centre, as seen in PEPS and PPDK. Despite the observed homology between these enzymes, pyruvate is not a substrate for phosphorylation by RPH enzymes^23^.

An understanding of the structural and molecular basis underlying rifamycin modification by RPHs will clarify their catalytic mechanism, basis of antibiotic recognition, evolutionary origins and provide the framework for the generation of rifamycin analogues that are not susceptible to modification. It is critical to develop a better understanding of the global rifamycin resistome, so that we can successfully identify, predict and prepare for the acquisition of these determinants by pathogenic bacteria. Here we provide structural and molecular insights into this unusual antibiotic resistance enzyme.

## Results

### The structure of RPH-*Lm* reveals a three-domain architecture

**Figure 1:**
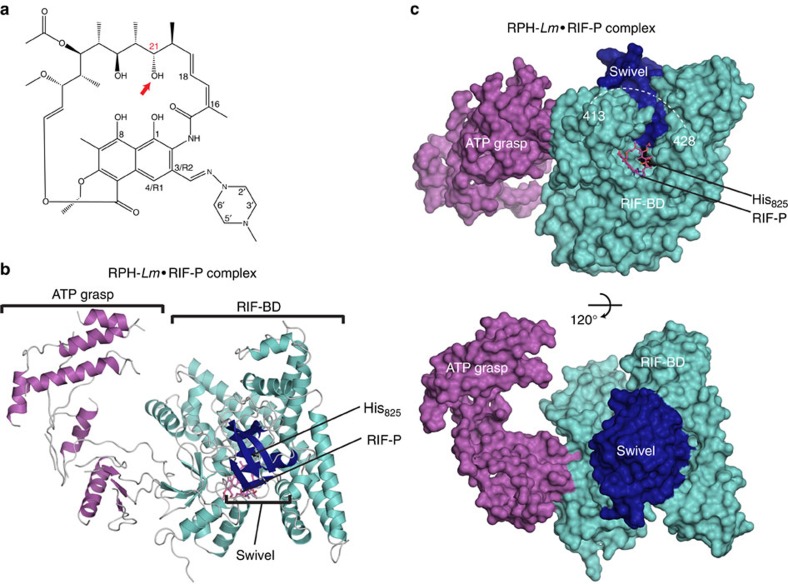
Structure of the RPH-*Lm*·RIF-P complex. (**a**) Chemical structure of RIF; the site of phosphorylation by RPH proteins is shown in red and indicated with an arrow. (**b**) Structure of the RPH-*Lm*·RIF-P complex. ATP-grasp (residues 1–314), RIF-binding (RIF-BD; residues 327–754) and swivel phosphohistidine (residues 767–865) domains are shown in shades of purple, cyan and dark blue, respectively. RIF-P is shown in pink sticks. (**c**) Surface representation of the RPH-*Lm*·RIF-P complex, the bottom view is rotated ∼120° from the top view. Dashed lines indicate a large disordered region between residues 413 and 428 in the RIF-BD. RIF-P is shown in pink sticks and the catalytic histidine labelled (His_825_).

We determined the crystal structures of three binary complexes of RPH from *L. monocytogenes* (RPH-*Lm*): RPH-*Lm*·Mg^2+^·ADP, RPH-*Lm*·RIF and RPH-*Lm*·RIF-phosphate (inactivated RIF; RIF-P). We first determined the structure of selenomethionine-derivatized RPH-*Lm*·RIF by the single anomalous dispersion method and used it as a model to solve the structures of native RPH-*Lm*·Mg^2+^·ADP and RPH-*Lm*·RIF-P by molecular replacement (statistics are shown in Table 1). The structure of RPH-*Lm* reveals that the enzyme features a three-domain architecture including an N-terminal ATP-binding domain followed by a large central domain that binds RIF (the RIF-binding domain/RIF-BD), and a smaller C-terminal domain (Fig. 1b and Supplementary Fig. 1).

**Figure 2:**
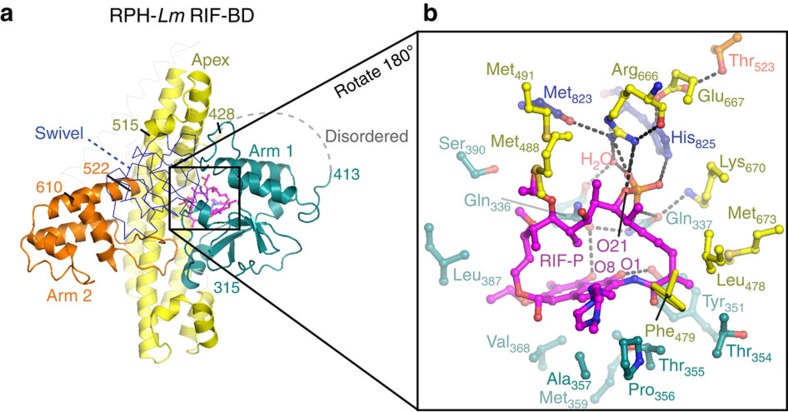
Multi-subdomain structure of the RPH-*Lm* RIF-BD and the RIF-binding site. (**a**) Three-dimensional structure of the RIF-BD showing subdomains. Apex (residues 428–505 and 648–693), arm 1 (residues 314–338, 357–413 and 738–753) and arm 2 (residues 522–580 and 594–611) subdomains are coloured in yellow, cyan and orange, respectively. Swivel phosphohistidine domain is shown in thin lines above the RIF-BD. Dashed line shows the disordered region between residues 413 and 428. RIF-P is shown as purple sticks. (**b**) Zoom of the RIF-P-binding site, rotated 180° from **a**. Residues forming hydrophobic or hydrogen bonding interactions with RIF-P, or with other residues comprising the binding site, are shown in sticks and the bound water is shown as a red sphere. Dashes indicate hydrogen bonds.

The ATP-binding domain (residues 1–314) has a fold characteristic of ATP-grasp enzymes^32^. The ATP-grasp fold is comprised of two α/β domains that simultaneously bind an ATP molecule between them (Supplementary Fig. 1). The RPH-*Lm* ATP-grasp domain closely resembles (Dali Z-score of 30.9) the structure of the ATP-grasp domain from a putative PEPS enzyme (PDB ID: 2OLS). Conversely, the RIF-BD (residues 327–754) adopts a rough cross shape that can be divided into three subdomains (Fig. 2a). One axis of the cross is formed by the apex subdomain, which is an α-helical region comprised of four helices (Fig. 2a). The other two subdomains form the axis perpendicular to the apex and are comprised of an α/β domain arranged into a two-layer sandwich (arm 1) and an orthogonal bundle of four α-helices (arm 2, Fig. 2b). One connection between the apex and arm 1 subdomains is disordered; notably, this region forms a ‘cap' over the RIF-binding site (Fig. 2a). Finally, the swivel phosphohistidine domain (residues 767–865) consists of a three-layer β/β/α sandwich (Fig. 1b), a fold characteristic of the swivel phosphohistidine domain of PPDK^33^. The swivel phosphohistidine domain is docked at the centre of the RIF-BD cross and makes contacts with all three subdomains.

Overall, RPH-*Lm* has an elongated shape with the ATP-grasp and RIF-binding domains defining the two ends of the molecule. Notably, in the absence of ADP, the ATP-grasp domain is largely disordered and in the absence of RIF, regions of the RIF-BD are disordered. These observations suggest significant mobility within regions of the domains in the absence of substrates. Furthermore, the swivel phosphohistidine domain must mediate crosstalk between the ATP-grasp and RIF-binding domains. However, in all three of the structures, this domain is bound to the RIF-BD (the ‘RIF-BD-engaged conformation') (Fig. 1b).

### RIF binds in a cleft between three subdomains of the RIF-BD

The search for structural homologues of the RPH-*Lm* RIF-BD domain did not reveal any candidates. RIF binds in a cleft formed between the intersection of the apex and arm 1 subdomains and the swivel phosphohistidine domain (Fig. 2a). The interaction between RPH-*Lm* and the antibiotic substrate is primarily hydrophobic, with one face of the naphthoquinone moiety of the antibiotic (Fig. 1a) cradled by a cluster of hydrophobic amino acids (Pro_356_, Ala_357_, Met_359_, Val_368_ and Leu_387_). The ansa-chain at position C(16)-C(18) is oriented by hydrophobic interactions with Leu_478_, Phe_479_, Met_673_ and the non-polar regions of Thr_354_ and Tyr_351_ side chains. RPH-*Lm* forms only two hydrogen bonds with the antibiotic: Gln_336_ with the C(8)-OH and Tyr_351_ with the C(1)-OH of the naphthoquinone ring, with the position of Gln_336_ stabilized by a hydrogen bond to Gln_337_.

The R1 and R2 groups of rifamycins (Fig. 1a), which are sites of medicinal chemical expansion in these drugs^17^,^23^, do not form significant interactions with RPH-*Lm*, with the exception of hydrophobic interactions between the R2 4-methyl-1-piperazinaminyl group and Phe_479_ (Fig. 2b). This observation rationalizes the ability of RPH-*Lm* to inactivate a broad selection of natural product and clinically utilized semi-synthetic rifamycins^23^.

The RPH-*Lm*·RIF and RPH-*Lm*·RIF-P structures are essentially identical in conformation (r.m.s.d. of 0.54 Å over all Cα atoms), but the interface between the RIF-BD and swivel phosphohistidine domains is more intimate in the presence of RIF. This observation implies that the interaction between the RIF-BD and the swivel phosphohistidine domain is responsive to the presence of antibiotic, which is consistent with the ligand-dependent degrees of disorder in the RPH structures, and suggests that the swivel phosphohistidine domain could disengage from the RIF-BD.

The phosphate group of RIF-P is deeply wedged into the RIF-BD's active site, stabilized by a distributed hydrogen-bonded network (Fig. 2b). This network features the single interaction between the swivel phosphohistidine domain of RPH-*Lm* and the drug: the catalytic His_825_ that carries the phosphate group is hydrogen-bonded to Glu_667_, which is concurrently hydrogen-bonded to Thr_523_ (from the arm 2 subdomain). Of the remaining RIF-P phosphate oxygens, one is bound to the side chain of Arg_666_. The final phosphate oxygen of RIF-P is held by an interaction with Gln_337_, which is hydrogen-bonded to Gln_336_ and Lys_670_.

### Two distant RPH-Lm active sites catalyse RIF inactivation

In our RPH-*Lm* structures, the β-phosphate of ADP and the phosphate on RIF-P are 49 Å apart, implying a significant conformational movement involved in RPH-*Lm* catalytic activity. We mutated a set of amino acids forming interactions with ADP or RIF, and assayed the subsequent enzyme variants *in vitro* and *in vivo* to investigate their roles within these two remotely located active centres. We focused our attention on substitution of amino acids that stabilize the polyphosphate moiety of the nucleotide, since correct positioning of this region will be critical for phospho-transfer to the catalytic His_825_. Mutagenesis of Arg_117_ and Thr_136_ (Supplementary Fig. 1b), which interact with the α-phosphate of ADP, resulted in complete loss of RIF resistance when the *rph-Lm* gene was overexpressed in a heterologous *Escherichia coli* host (Table 2). Interestingly, these variants retained ATP pyrophosphatase activity *in vitro*, with rates comparable to wild-type RPH-*Lm* (Table 2). In contrast, the mutant enzymes were not competent in catalysing the second half of the reaction (Table 2) and subsequent analysis utilizing liquid chromatography with mass spectrometry detection (LC/MS) revealed an absence of RIF-P formation (Supplementary Table 1). Overall, these two variants were catalytically less efficient with a 10-fold reduction in the *k*_cat_*/K*_m_ value (Table 2). We next mutated Lys_22_ that interacts with both the α- and β-phosphates of ADP (Supplementary Fig. 1b); this position in PPDK (also Lys_22_) has been previously implicated in interacting with the γ-phosphate of ATP^34^ and may play a role in γ-phosphate binding by RPH-*Lm*. Substitution of Lys_22_ to Ala resulted in complete loss of RIF resistance (Table 2) and no enzyme activity was observed *in vitro* (Table 2 and Supplementary Table 1). Finally, in the RPH-*Lm*·Mg^2+^·ADP structure, Glu_297_ appears to be coordinating the catalytic Mg^2+^ ion (Supplementary Fig. 1b). However, replacement of this residue for Ala had no effect on RIF resistance *in vivo* and the mutant enzyme was catalytically competent towards both reactions in the ATP-grasp and the RIF-BD (Table 2 and Supplementary Table 1). Surprisingly, this RPH variant demonstrated 10-fold higher rate of substrate turnover (*k*_cat_) compared with wild-type enzyme (Table 2). Nevertheless, due to an increase in the substrate *K*_m_, the overall catalytic efficiency (*k*_cat_*/K*_m_) was comparable to that of wild type. Since Mg^2+^ ought to be tightly bound to ATP, these results reveal that Mg^2+^ coordination by Glu_297_ is not necessary for catalysis.

To interrogate the role of residues participating in RIF recognition, we next generated a further five substitutions within the RIF-BD. We substituted Val_368,_ which is one of a cluster of amino acids cradling the napthoquinone region of RIF via hydrophobic interactions (Fig. 2b). Substitution of this residue for Thr or Glu resulted in a two- to fourfold decrease in RIF resistance and an increase in the RIF *K*_m_ by six- and eightfold (Table 2). Subsequent steady-state kinetic analysis of these enzyme variants revealed surprising values for the rate of substrate conversion (*k*_cat_). Rather than a decrease in the catalytic efficiency as anticipated from the decrease in RIF resistance, we observed a 10-fold increase in the *k*_cat_*/K*_m_ for ATP (Table 2) with both Thr and Glu substitutions. This finding highlights the complexity of this enzyme's catalytic cycle and could imply that affecting binding of RIF favours the first phosphotransfer reaction (from ATP to the catalytic His_825_) over the second (transfer to the antibiotic). To investigate the importance of hydrogen bond formation between RPH-*Lm* and the napthoquinone ring, we substituted Tyr_351_ for Phe. However, this substitution had little effect on enzyme activity. Susceptibility levels, kinetic values and RIF-P formation were comparable to wild type, showing that hydrophobic interactions outweigh that of hydrogen bond formation. Next we probed residues involved in the hydrogen-bond network formed between RIF-P and RPH-*Lm*. Glu_667_ and Arg_666_ appear to interact directly with the phosphate of RIF-P and their substitution to Ala abolished all RIF inactivation by RPH-*Lm* (Table 2 and Supplementary Table 1). However, similar to the Arg_117_Ala and Thr_136_Val substitutions generated in the ATP-grasp domain, these variants were still able to catalyse the partial reaction within the N-terminal ATP-grasp domain (with a 10-fold reduction in *k*_cat_*/K*_m_ values), but were not capable of catalysing the second half of the reaction since no RIF modification was detected (Table 2 and Supplementary Table 1). Finally, we substituted Gln_337_ that interacts with one of the RIF-P phosphate oxygen. Replacement of this residue with Ala resulted in a fourfold decrease in RIF resistance (Table 2), however, steady-state kinetics were comparable to that of wild type, indicating that this residue's interaction with the phosphate of the inactivation product is not central to catalysis. Overall, these results clearly implicate the Glu_667_-Arg_666_ sequence as being critical for the second phosphotransferase reaction by RPH.

This mutagenic approach reveals that there are two distinct stages to the catalytic activity of RPH-*Lm*. Residues residing within the ATP-grasp are implicated in correct positioning of the ATP α-β phosphodiester bond (Lys_22_, Arg_117_ and Thr_136_) for the first phosphotransferase reaction and residues of the RIF-BD apex subdomain (Glu_666_ and Arg_667_) are critical determinants for the second phosphotransferase reaction.

### Catalysis is facilitated via a swivelling mechanism

Given that the two active centres of RPH-*Lm* and their key catalytic residues are spatially distant, it is clear that in order for His_825_ to become phosphorylated, the swivel phosphohistidine domain must localize to the ATP-grasp domain. This phosphorylated enzyme intermediate must then transition to the RIF-BD. We took advantage of solution small-angle X-ray scattering (SAXS) and analysed the behaviour of RPH-*Lm* in a ligand-dependent manner. All scattering data for RPH-*Lm* (as the apoenzyme, or in various ligand-bound states) were of excellent quality and indicate that the enzyme behaves as monomers in solution (Supplementary Table 2). *Ab initio* modelling of the RPH-*Lm* scattering curve results in models resembling the one captured by the crystal structures (Supplementary Fig. 2). However, comparison of theoretical scattering curves calculated from the crystal structures and the experimental scattering data resulted in poor fits (χ^2^ values ranging 1.6–2.9) as judged by CRYSOL^35^. Therefore, we entertained the possibility that the scattering curve of RPH-*Lm* represented the average scattering of multiple conformations of the swivel phosphohistidine domain.

To test this hypothesis, we analysed the merged scattering curves of RPH-*Lm* (apo), RPH-*Lm*·Mg^2+^·ADP and RPH-*Lm*·RIF, using the ensemble optimization method (EOM)^36^,^37^. This analysis confirmed that the swivel phosphohistidine domain of RPH-*Lm* is mobile (χ^2^ ranging from 1.0–1.3), but not completely flexible because the EOM sub-ensembles were populated by only three conformations: RIF-BD-engaged, ATP-grasp engaged and transient (Table 3). The distribution among the three conformations, however, varies depending on the ligands bound to the enzyme. The sub-ensemble that best describes the scattering data of the RPH-*Lm* in the apoenzyme state is composed of two conformations that are nearly equally populated (Table 3). Visual inspection of the conformations defining the sub-ensemble indicates that the preferred ‘ground state' of the swivel phosphohistidine domain, in the absence of ligands, is bound to the RIF-BD, with a significant population in the transient state (Table 3). The sub-ensemble describing the RPH-*Lm*·RIF scattering data had an increased proportion of the population in the RIF-BD-engaged, reflecting the stabilizing effect of this ligand. Notably, 30% of the RPH-*Lm*·RIF population was in the ATP-grasp engaged state, revealing that the presence of RIF restricts the conformational flexibility of the enzyme and enhances transition of the swivel phosphohistidine domain towards the ATP-grasp domain. Binding of nucleotide also restricts the conformational flexibility of the enzyme, with the sub-ensemble describing the scattering curve of the RPH-*Lm*·Mg^2+^·ADP sample still dominated by the population adopting the RIF-BD-engaged state and a similar proportion in the ATP-grasp engaged state, but including a small proportion in the transient state (Table 3). We interpret this result to reflect that ADP is not a substrate for the enzyme as it lacks the γ-phosphate that the enzyme recognizes; the ATP-grasp engaged state might only be significantly populated in the presence of nucleotide capable of transferring a pyrophosphate moiety to the protein.

We sought to monitor the conformation of the swivel phosphohistidine domain using non-hydrolysable analogues of ATP that arrest the reaction at specific points during the reaction. We measured the scattering curves of RPH-*Lm* bound to Mg^2+^·AMPPnP and Mg^2+^·AMPcPP in the absence and presence of RIF. By monitoring pyrophosphatase activity, we verified that RPH-*Lm* was unable to hydrolyse these analogues. RPH-*Lm* bound to either analogue showed an increase in the ATP-grasp engaged population, indicating stabilization of this particular domain interface (Table 3). With RIF and Mg^2+^·AMPcPP, the EOM analysis did not identify any swivel phosphohistidine domain bound to the ATP-grasp domain, and actually resulted in an increase in the proportion of the swivel phosphohistidine domain engaged to the RIF-BD, to the highest proportion observed in our experiments. Conversely, the RIF-Mg^2+^·AMP-PnP analysis showed 60% of the population with the swivel phosphohistidine domain engaged to the ATP-grasp domain. These results illustrate that recognition of nucleotide substrates by RPH-*Lm* is dependent on oxygen at the α-β phosphodiester bond. This is consistent with our RIF-Mg^2+^·ADP crystal structure showing an interaction between Mg^2+^ and the α-β phosphodiester oxygen (Supplementary Fig. 1b), and is also consistent with our previous results depicting transfer of the β-phosphate from ATP to RIF^23^.

### RPH-Lm structural similarities and ancestral origins

**Figure 3:**
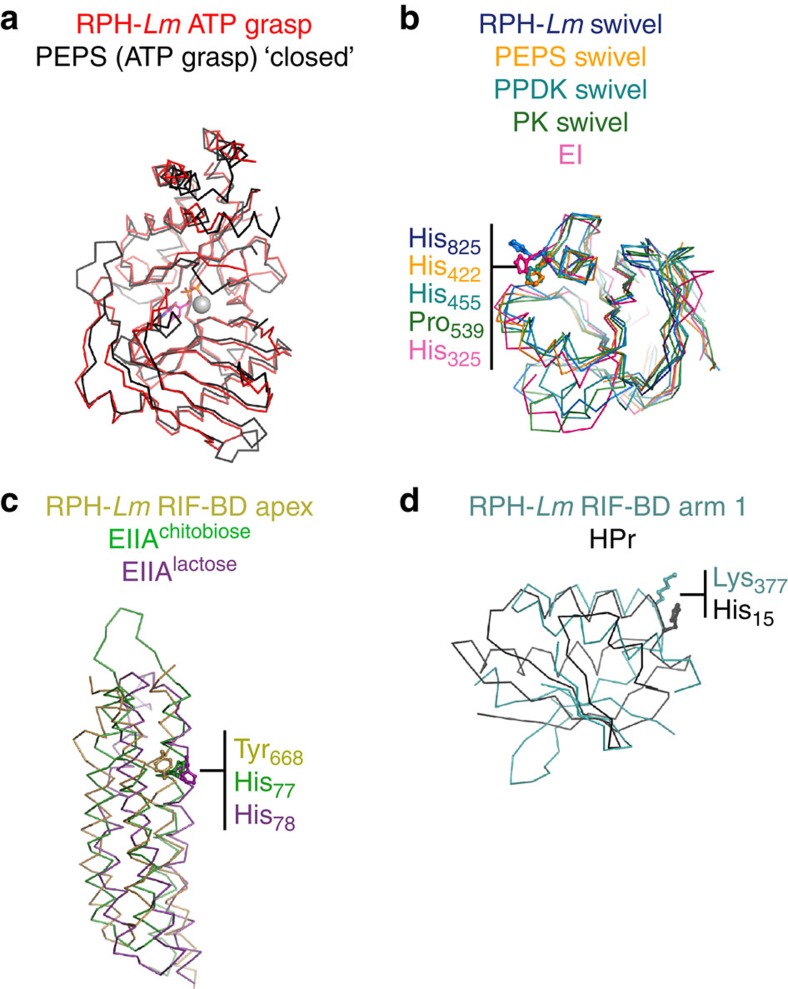
RPH-*Lm* shows structural similarity with phospho-relay proteins including the PTS. (**a**) Comparison of the ATP-grasp domains of RPH-*Lm* and PEPS (PDB 2OLS); r.m.s.d. 1.7 Å (275 Cα), 33% identity. (**b**) Comparison of the swivel phosphohistidine domains of RPH-*Lm* with PEPS (PDB 2OLS), PPDK (PDB 1D1K), pyruvate kinase/PK (PDB 3T07) and the phosphohistidine carrier domain of the PTS system EI protein (PDB 3EZB); r.m.s.d. 1.3–2.1 Å (82–101 Cα), 24–43% identity. (**c**) Comparison of the apex subdomain of the RPH-*Lm* RIF-BD with PTS system EIIA proteins (EIIA^chitobiose^, PDB 2LRK and EIIA^lactose^, PDB 1E2A); r.m.s.d. 3.3 Å (91 Cα), 3% identity. (**d**) Comparison of the arm 1 subdomain of the RPH-*Lm* RIF-BD with the PTS system HPr protein (PDB 3EZB). Side chains shown in sticks are the catalytic histidines from their respective proteins, and their equivalents from their structural homologues; r.m.s.d. 3.6 Å (38 Cα), 8% identity.

Given the structural novelty of RPH and the complexity of its antibiotic inactivation mechanism, we were interested in identifying the ancestral origins for this enzyme through structural comparisons within the PDB. The RPH-*Lm* ATP-grasp domain is structurally indistinguishable from PEPS (sequence identity 43%) (Fig. 3a). Structural homology searches reveal that the RPH-*Lm* swivel phosphohistidine domain is similar to PPDK, pyruvate kinase (PK) and EI of the sugar phosphotransferase system (PTS) (Fig. 3b and Supplementary Fig. 3)^38^,^39^. The PTS is a bacterial transport system that comprises a phospho-relay system that transfers the phosphate group from PEP to histidine residues on enzyme I (EI), histidine phosphocarrier (HPr), enzyme IIA (EIIA) and the enzyme IIC (EIIC) proteins, in a cascade-like fashion^40^. Unexpectedly, the three RIF-BD subdomains show weak yet significant structural similarity with individual components of the PTS. The apex subdomain of RPH-*Lm* showed similarity with EIIA enzymes (Fig. 3c) and the arm 1 subdomain of RPH-*Lm* is similar to the HPr protein (Fig. 3d).

Notably, the only active site similarity between RPH-*Lm* and the PTS was in the conservation of the histidine situated within the swivel phosphohistidine domains of RPH-*Lm*, PEPS, PPDK and EI (His_825_, His_422_, His_425_ and His_325_, respectively, Fig. 3b). These observations indicate that RPH-*Lm* shares common ancestors with components of glycolytic enzymes that have evolved in a novel arrangement to form its catalytic site. RPH-*Lm* can be thought of as a chimera of various components of glycolytic phospho-relay systems involving pyruvate and small sugars. Such a complexity of molecular assembly is unprecedented in antibiotic resistance proteins.

### RPH is part of a larger superfamily of phosphotransferases

**Figure 4:**
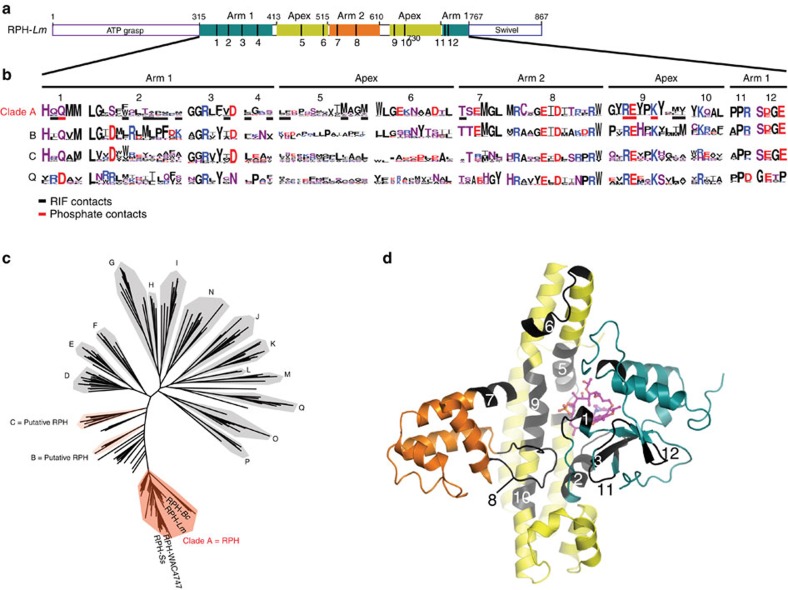
Tracking the RIF-BD and RIF-binding site across RPH homologues. (**a**) Primary structure representation of the RIF-BD subdomain structure. The apex, arm 1 and arm 2 subdomains are coloured yellow, green and orange, respectively. Twelve key RIF-BD sequence motifs identified in the sequence and/or structure of RPH-*Lm* are indicated with vertical bars and numbering. (**b**) Sequence logos for 12 sequence motifs of the RIF-BD in clades A, B, C and Q identified in phylogenetic reconstruction in **c**. Height of the amino acid letter indicates higher conservation within that clade. Colouring of amino acids: R/K (basic)=blue; D/E (acidic)=red; Q/N/S/T/H/C (polar)=purple; and L/I/V/W/F/Y/M/A/P (hydrophobic)=black. Black and red bars underneath the logos represent those amino acids identified to interact with the rifamycin scaffold and phosphate group, respectively, as identified in the RPH-*Lm*·RIF-P complex crystal structure. (**c**) Phylogenetic reconstruction of 650 sequence homologues of RPH-*Lm* identified in Genbank. Clade A contains the experimentally validated^23^ RIF resistance enzymes RPH-*Lm*, RPH-*Bc,* RPH-WAC4747 and RPH-*Ss*, which are shaded in red. Clades B and C, which according to our sequence analysis include putative RPH enzymes, are shaded in light red. (**d**) RPH-*Lm* RIF-BD, coloured and numbered according to **a** and **b**.

We were interested in tracing the distribution of RIF-inactivating enzymes. We reasoned that the multi-subdomain architecture of the RIF-BD and the RIF-binding site should be conserved for RIF inactivation. Phylogenetic reconstruction of RPH enzymes and homologues, present in both bacteria and archaea, identified multiple discrete clades (Fig. 4c). One monophyletic set (clade A; >42% identical with RPH-*Lm*) includes the experimentally-validated RPH enzymes RPH-*Lm*, RPH-*Bc*, RPH-WAC4747 and RPH-*Ss* (ref. 23). Clade A sequences conserve the RIF-BD three-subdomain architecture (Fig. 4a,b) and further analysis reveals 12 conserved sequence signatures within this region (Fig. 4d). These blocks localize to key regions of the RIF-BD, including the RIF-binding site, interfaces between the RIF-BD subdomains or ATP-grasp and swivel phosphohistidine domain interfaces. The high conservation of these residue signatures across clade A implies that the protein sequences within this group adopt a similar overall architecture and substrate specificity.

We next investigated how far RIF-specific sequence signatures extend along our phylogeny of putative RPH homologues (Fig. 4c). Analysis revealed that the arms 1 and 2 subdomains are more conserved than the apex subdomain (Fig. 4b). Key motifs identified in clade A could also be identified in clades B and C, including persistence of RIF-interacting residues. Residues that interact with the RIF-P phosphate, including a Gln in block 1 and the Arg/Glu/Lys residues in block 9, are highly conserved. Since RIF-interacting residues are conserved, we predict clades B and C are also candidate rifamycin inactivating enzymes (these clades include sequences from *Peptococcaceae, Clostridiaceae, Haloferacaeae* and *Natrialbaceae*).

We were not able to identify most of the sequence blocks and/or RIF-interacting residues in representatives of Clade Q, occupying the far end of the phylogenetic reconstruction relative to clade A. However, clade Q did conserve the catalytic Arg-Glu and Lys residues found within block 9 (Fig. 4b). Based on these observations, we anticipate that representatives of distant clades within the phylogenetic reconstruction retain general phosphotransferase activity, but their substrate specificity has significantly diverged from that of RIF-specific enzymes. Indeed, we heterologously expressed one protein from clade N of our tree (*Clostridium difficile,* Genbank ID: FN538970.1, Fig. 4c) in *E. coli* and confirmed that this enzyme does not confer RIF resistance.

## Discussion

This study enhances the growing body of evidence highlighting the importance of proto- and silent-resistance in the global challenge of antibiotic resistance. While numerous examples of antibiotic kinases have been reported, our structural and molecular studies of RPH reveal that these enzymes are distinct from any known antibiotic kinase and therefore represent a class of enzyme with an unusually wide distribution in bacterial genomes, and a complex catalytic mechanism.

**Figure 5:**
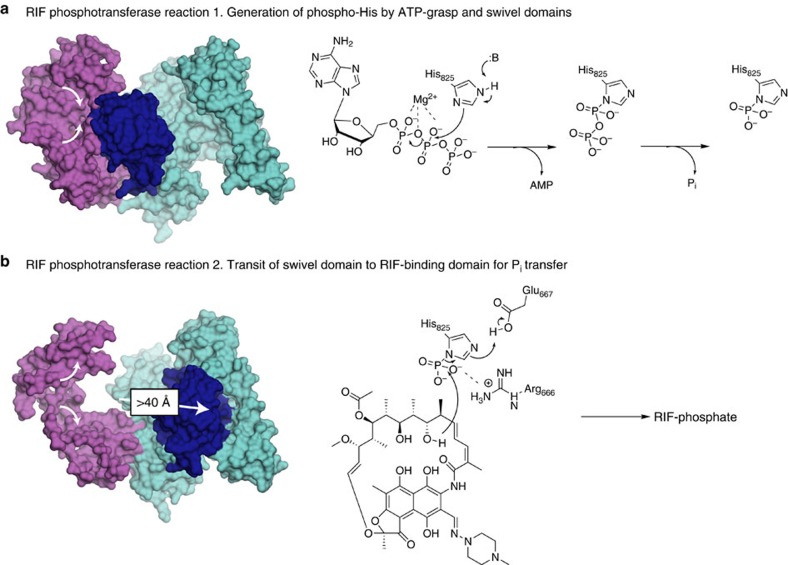
Enzymatic mechanism of RPH-*Lm.* The two distinct phosphotransferase reactions are shown. (**a**) Reaction 1, which takes place in the ATP-grasp domain (coloured purple) and involves attack of the ATP β-phosphate by His_825_ of the phosphohistidine swivel domain (coloured dark blue), forming a pyrophosphorylated histidine residue. Water hydrolyses the γ-phosphate, leaving phosphohistidine; this reaction is followed by transit (>40 Å distance) of the swivel phosphohistidine domain to the RIF-BD (coloured cyan). The white arrows depict the conformational changes observed in the ATP-grasp domain. (**b**) Reaction 2, which takes place in the RIF-BD. The C(21)-hydroxyl of RIF attacks the phosphorylated His_825_ that is positioned by Arg_666_ and Glu_667_. This yields the RIF-phosphate product and the phosphohistidine RPH-*Lm* adopts the transient conformation until binding of the next ATP molecule.

Crystallography, small-angle scattering, mutagenesis and enzyme characterization showed that the catalytic basis of RIF modification is complex, involving a two-stage mechanism, with transition of a small C-terminal swivel phosphohistidine domain between two active centres. Our combined structural and biochemical data allows for a proposed model depicting the molecular mechanism of RPH (Fig. 5). The enzymes conformational flexibility appears to be a response to ligand binding. In the absence of substrates, the swivel phosphohistidine domain is either transient or docked to the RIF-BD. Upon binding of nucleotide, we begin to observe a larger population of molecules adopting the ATP-grasp engaged state. Similar to the presence of nucleotide, binding of RIF also results in a larger proportion of molecules adopting the ATP-grasp engaged state, which we interpret as ligand-induced restriction of the intrinsic conformational flexibility of the enzyme, enhancing transition of the swivel phosphohistidine domain toward the ATP-grasp domain. Indeed, there is a significant increase in the population of swivel·ATP-grasp domain engaged conformations when both ligands are present (Table 3). We hypothesize that the molecular basis underlying this observation is the conformational change induced by nucleotide binding in the ATP-grasp domain. Indeed, binding of ATP to the ATP-grasp domain of PPDK has previously been shown to induce a so-called ‘grasping' onto the nucleoside triphosphate^29^,^30^,^31^. This regional conformational change creates a binding surface for interaction with the swivel phosphohistidine domain. In all three of our structures, this domain is in the same conformation/state. Since PPDK has been observed to transition between ‘open'/ATP-free and ‘closed'/ATP-grasped states^31^,^33^, structural comparisons between RPH-*Lm* and the two forms of PPDK reveal that our crystal structures correspond to the closed state (Fig. 3a). Thus, by analogy with PPDK, the ATP-grasp domain of RPH-*Lm* likely undergoes a ‘grasping' conformational change onto ATP, which creates a binding surface for the swivel phosphohistidine domain. The ‘grasping' could also induce a conformational change elsewhere in RPH-*Lm*, to facilitate transit of the swivel phosphohistidine domain to the ATP-grasp domain; such conformational changes were observed in a linker region of PPDK^41^.

The polyphosphate moiety of ATP is positioned by a subset of amino acids, with Arg_117_ and Thr_136_ implicated in binding the α-phosphate, an observed interaction between Mg^2+^ and the α-β phosphodiester oxygen and coordination of the tri-phosphate by Lys_22_. Binding of the ATP γ-phosphate could stabilize regions of the ATP-grasp domain that are unresolved in our crystal structures and are appropriately located to participate in binding of the swivel phosphohistidine domain. Engagement of the swivel phosphohistidine domain to the ATP-grasp domain would bring the N^ɛ^ atom of His_825_ into vicinity of the polyphosphate moiety. Coordination of Mg^2+^ by the α-β phosphodiester oxygen increases electrophilicity of the phosphates, facilitating nucleophilic attack of the β-phosphate by the N^ɛ^ atom of His_825_. In turn, this would liberate the β- and γ-phosphates from ATP forming pyrophosphorylated His_825_, the γ-phosphate of which is then hydrolysed resulting in a β-phosphorylated enzyme intermediate.

Completion of the first phosphotransferase reaction would destabilize the swivel-docking site on the ATP-grasp domain, resulting in transition of the phosphohistidine swivel phosphohistidine domain to the RIF-BD (Fig. 5). This preferred ‘ground-state' of the enzyme brings the phosphohistidine into proximity of the C(21)-OH of RIF. Our study revealed a key set of amino acids (Arg_666_, Glu_667_ and Lys_670_) involved in formation of a hydrogen-bonded network between RIF-P and RPH-*Lm*. We envisage that Glu_667_ stabilizes the position of His_825_ such that its bound phosphate interacts with Arg_666_. An active site base would be predicted to deprotonate the RIF hydroxyl at C(21) to accept the phosphate. A scan of nearby residues does not identify a reasonable candidate. This may be the result of conformational changes in the enzyme during catalysis that would position an active site base correctly or the position of Arg_666_ may stabilize a meta-phosphate-like transition or intermediate state that would not require the participation of a RIF deprotonating base. The phosphate is directly transferred to the positioned C(21)-OH, liberating His_825_ for further rounds of phosphotransfer, and the swivel phosphohistidine re-adopts its transient/mobile conformational state.

There is a clear similarity between RPH enzymes and proteins involved in the transport and modification of sugars in bacteria. RPH-*Lm* appears to be a chimera of several components of phospho-relay systems involving pyruvate and small sugars. This finding provides insight into the evolution of this resistance determinant from proteins that are not directly involved in antibiotic resistance. Indeed, to our knowledge, this form of multi-domain recruitment is new to antibiotic resistance. However, it is well documented that during evolution, proteins are produced with new functions through the process of gene combination, duplication and sequence divergence^42^,^43^,^44^. Indeed, this appears to be the evolutionary path of the RPH enzymes.

The RPH enzymes are diverse and widespread, with orthologs identified in both bacteria and archaea, and even in microorganisms susceptible to rifamycins. However, following phylogenetic reconstruction and analysis of the RIF-BD conservation, it is clear that not all members of this protein family that we previously identified^23^ are RIF modifying enzymes. The sequences of the intervening region (equivalent to the RIF-BD of RPH-*Lm*) and the RIF-binding site rapidly diverge over the tree/course of evolution, while the sequences of the ATP-grasp and swivel phosphohistidine domains are relatively conserved. This finding indicates that general phosphotransferase activity may be conserved across RPH homologues, but the specificity of these enzymes has diverged to substrates other than RIF. Along these lines, the RIF-specific proteins may have evolved from ancestors that were primarily active on other small molecules. Thus our phylogenetic analysis positions this collection of enzymes as a new widespread protein family of small molecule dikinases; it is clear that at least the members of the RPH-containing clade A function on antibiotics. Future structural studies are warranted to elucidate the substrate specificity of these enzymes to better understand this antibiotic proto-resistome.

## Methods

### Purification of RPH-Lm for crystallization

The *rph-Lm* sequence from *L. monocytogenes* str. 4b F2365 was amplified by PCR and subcloned into the pMCSG53 expression vector that codes for a N-terminal His_6_-tag and a TEV protease cleavage site. RPH-*Lm* was expressed in *E. coli* BL21(DE3) codon plus cells, grown to an OD600 of 0.6 at 37 °C, chilled to 16 °C and induced overnight with 500 μM isopropyl β-D-thiogalactopyranoside. Se-Met-substituted RPH-*Lm* was expressed using the standard M9 high-yield growth procedure according to the manufacturer's instructions (Shanghai Medicilon). Cells were harvested via centrifugation at 5,000*g* and pellets resuspended in binding buffer (50 mM Hepes (pH 7.5), 100–300 mM NaCl, 10 mM imidazole and 2% glycerol (v/v)), lysed by sonication, and cell debris removed via centrifugation at 30,000*g*. Cleared lysate was loaded onto a 5 ml Ni-NTA column (QIAGEN) pre-equilibrated with binding buffer, extensively washed with binding buffer containing 30 mM imidazole, and protein was eluted using the above buffer with 250 mM imidazole. His_6_-tag was removed by cleavage with TEV protease overnight at 4 °C in dialysis buffer (0.3 M NaCl, 50 mM Hepes (pH 7.5), 5% glycerol and 0.5 mM tris[2-carboxyethyl]phosphine), followed by binding to Ni-NTA resin and capture of flow through. Fractions containing RPH-*Lm* were identified by SDS–polyacrylamide gel electrophoresis and further purified via gel filtration on a HiLoad 16/60 Superdex75 prep-grade column (10 mM Hepes (pH 7.5) and 50 mM KCl).

### Crystallization of RPH-Lm and data collection

All crystallizations were performed at room temperature using the sitting drop method and 0.5 μl protein or protein:ligand mixture plus 0.5 μl reservoir solution. To obtain the RPH-*Lm*·RIF complex, 20 mg ml^−1^ Se-Met RPH-*Lm* was crystallized with 5 mM RIF and reservoir (0.1 M SPG buffer pH 6, 25% (w/v) PEG3350). The crystal was cryoprotected in 10% MPD and paratone oil. To obtain the RPH-*Lm*·Mg^2+^·ADP complex, 20 mg ml^−1^ native RPH-*Lm* was crystallized with 5 mM ADP and reservoir solution (20% (w/v) PEG3350, 0.2 M calcium chloride, 2% (w/v) glycerol). The crystal was cryoprotected in glycerol and paratone. To obtain the RPH-*Lm*·RIF-P complex, 20 mg ml^−1^ native RPH-*Lm* was crystallized with 5 mM RIF and 5 mM ATP with reservoir solution (35% tacsimate and 10 mM potassium chloride). The crystal was cryoprotected in 10% MPD.

All X-ray diffraction data at 100 K were collected at the Advanced Photon Source, Argonne National Laboratory, Life Sciences Collaborative Access Team beamline 21 (for the Se-Met-substituted RPH-*Lm*·RIF complex, at the selenium absorption peak). X-ray data were reduced with HKL-3000 (ref. 45) or XDS^46^ and CCP4 Aimless^47^. The structure of the Se-Met-substituted RPH-*Lm*·RIF complex was solved by single anomalous dispersion using Phenix.solve^48^ that identified 23 out of 25 selenium sites in the asymmetric unit (one protein molecule). An initial model of the protein was built using Phenix.autobuild, followed by manual model building and refinement with Coot^49^ and Phenix.refine. Structures of the RPH-*Lm*·Mg^2+^·ADP and RPH-*Lm*·RIF-P complexes were solved by molecular replacement using the RPH-*Lm*·RIF structure as a search model in Phenix.phaser and refined using Phenix.refine and Coot. The presence of all ligand molecules were validated using omit maps: all atoms of the ligand plus other atoms within 5 Å of the ligand were deleted, followed by simulated annealing (Cartesian) using Phenix.refine and model building into residual *F*obs−*F*calc density. All B-factors were refined and TLS parameterization was included in final rounds of refinement. All geometry was verified using the Phenix and the wwPDB server. Regions of RPH-*Lm* were disordered in all three-crystal structures, including residues 53–55, 67–77, 121–130 and 165–172 of the ATP-grasp domain and residues 414–427 of the RIF-BD. For these reasons, along with the modest resolution limits of the X-ray diffraction data, we retained some regions of the RPH structures as unmodelled or with side chains truncated to the Cα atoms. Simulated annealing omit electron density maps for all ligands can be found in Supplementary Fig. 4.

### Structural analysis

Structure similarity searches were performed using Dali^50^, PDBeFold^51^ and Cath^52^,^53^. Interactions between enzyme and ligand atoms were identified with PyMOL or Coot. Sequence analysis was facilitated by Jalview.

### Site-directed mutagenesis

Site-specific amino acid substitution was performed using Phusion High-Fidelity DNA Polymerase (Thermo Scientific) and the construct pET19Tb:*rph-Lm* (ref. 23). Primers were designed using the QuikChange primer design program (Agilent Technologies). Selected mutants were confirmed by sequencing at the MOBIX Central Facility (McMaster University, Hamilton, Canada). Successful mutants were transformed into *E. coli* Rosetta(DE3)pLysS for susceptibility determination and protein over-expression.

### RIF susceptibility determination

RIF (Sigma-Aldrich, Oakville, ON) susceptibility of RPH-*Lm* variants was determined in duplicate, using the micro-dilution broth method, according to Clinical and Laboratory Standards Institute guidelines^54^, in the absence of isopropyl β-D-1-thiogalactopyranoside (IPTG).

### RPH-Lm *in vitro* studies of enzyme variants

*In vitro* activity of RPH-*Lm* and variants was determined by measurement of the liberation of inorganic phosphate, using the EnzChek Phosphate Assay Kit (Life Technologies, Burlington ON). A Spectramax Plus^384^ (Molecular Devices) microtitre plate reader was used to monitor the reactions at 360 nm. All reactions were performed in 96-well Nunc plates (Thermo Scientific), in duplicate, with a final volume of 100 μl, 11.3 μM RPH-*Lm* and 50 mM Hepes (pH 7.5), 5 mM MgCl_2_, 40 mM NH_4_Cl_2_. For characterization of ATP dependence, RIF was kept at five times the *K*_m_, and for RIF assessment, ATP was also kept at five times the *K*_m_. Before initiation of the assay, reactions were incubated in the plate reader, with shaking for 5 min at 25 °C. Nucleotide was added to initiate the reaction and inorganic phosphate production was monitored for 10 min. The GraFit 5.0.13 software suite (Erathacus Software) was used to assess the data.

For assessment of RPH-*Lm* hydrolysis of ATP analogues, AMP-cPP and AMP-PnP (Sigma-Aldrich, Oakville, ON), reactions were performed as above, with the analogues replacing ATP. RIF and nucleotide analogues were kept at five times that of their respective *K*_m_ values.

Quantification of RIF-phosphate production, using LC/MS, was performed using a QTRAP LC/MS/MS system (Applied Biosystems) with an electrospray ion source. Reactions proceeded for 1 h at 25 °C in a final volume of 100 μl, in buffer A (20 mM Hepes (pH 7.5), 100 mM KCl, 5 mM MgCl_2_ and 1 mM β-mercaptoethanol). Reactions consisted of 500 μM ATP, 25 μM RIF and 5 μg RPH-*Lm* variants. An equal volume of ice-cold methanol was used to terminate the reaction, 50 μl of which was analysed using LC/MS. Product peaks were integrated using Analyst software (Agilent).

### SAXS sample preparation, data collection and analysis

RPH-*Lm* was overexpressed in *E. coli* Rosetta(DE3) pLysS using the autoinduction method at 25 °C for 40 h. Samples were purified using a Ni-NTA column (QIAGEN), and were eluted in 50 mM Hepes (pH 7.5), 300 mM NaCl and 250 mM imidazole, followed by dialysis at 4 °C overnight in 50 mM Hepes (pH 7.5) and 300 mM NaCl. The His-tag was removed by cleavage with TEV protease at 12 °C for 16 h, the protein preparation was then applied to a second Ni-NTA column and the flow through collected. Cleaved RPH-*Lm* was then purified further using gel filtration chromatography with a HiLoad 26/60 Superdex 200 prep-grade column in buffer A. The purified protein was concentrated and stored at −80 °C with 20% (*v/v*) glycerol. Each SAXS samples was then prepared using an aliquot of this purified protein and a final purification step involving a Superdex 200 10/300 GL (GE Healthcare) gel filtration column equilibrated with buffer A. This column was calibrated using a gel filtration molecular weight marker kit for molecular weights ranging 12,000–200,000 Da (Sigma-Aldrich); RPH-*Lm* eluted at a volume of 12.5 ml, corresponding to 123 kDa (an elongated monomer).

Following the final gel filtration purification step, nucleotide and/or RIF were introduced (at 10 times the molar concentration of the protein) during a final concentration step intended to saturate the protein. The eluates from the concentrator were used as blanks. Sample homogeneity was confirmed by dynamic light scattering and scattering data were measured on a Rigaku BioSAXS-1000 instrument at 10 °C. Consecutive scans of 10, 30 and 120 min were collected over a range of protein concentration (2–6 mg ml^−1^). SAXSLab 3.0.0r1 (Rigaku) was used to generate the scattering curves. The lack of radiation damage was confirmed by comparing the scattering curves at the beginning and the end of data collection. Comparison and analysis of the scattering curves was done using the ATSAS 2.6.0 suite^55^. Samples were devoid of inter-particle interactions as judged from the Guinier plots, and folded as judged from the Kratky plots. Scattering curves were generated by merging the low q range from the most diluted samples with the higher q range from the most concentrated samples using the automerge tool in the ATSAS 2.6.0 suite^55^. Radius of gyration and pair-distance distribution functions were determined using Primus and GNOM (Supplementary Table S2)^55^. The reported molecular weights were calculated based on the volume of correlation^56^.

*Ab initio* modelling of the RPH-*Lm* scattering curve was performed using GASBOR^57^ and comparison of the theoretical scattering curves of the RPH-*Lm*·Mg^2+^·ADP and RPH-*Lm*·RIF crystal structures with CRYSOL^35^. The scattering curves for the rest of the RPH-*Lm* samples were analysed using the ensemble optimization method^36^,^37^ to determine the conformational flexibility. An ensemble of 10,000 random conformations were generated by keeping the nucleotide- and RIF-binding domains constant and allowing the swivel phosphohistidine domain (residues 764–863) to adopt any random conformation. EOM selected the optimized sub-ensembles from the random pool using a genetic algorithm (Table 3).

### Phylogenetic analysis

The phylogenetic reconstruction shown in Fig. 4c was previously performed on a set 670 protein sequences^23^. The clades in the tree (Fig. 4c) were chosen to reflect groups with high bootstrap support (>80) and were utilized for generation of sequence logos using the Weblogo server^58^.

## Additional information

**Accession codes:** the RPH-*Lm* crystal structures have been deposited in the PDB with accession codes 5FBS (RPH-*Lm*·Mg^2+^·ADP), 5FBT (RPH-*Lm*·RIF) and 5FBU (RPH-*Lm*·RIF-P).

**How to cite this article:** Stogios, P. J. *et al.* Rifampin phosphotransferase is an unusual antibiotic resistance kinase. *Nat. Commun.* 7:11343 doi: 10.1038/ncomms11343 (2016).

## Supplementary Material

Supplementary Information Supplementary Figure 1-4, Supplementary Tables 1-2 and Supplementary References.

## Figures and Tables

**Table 1:** X-ray diffraction data collection and refinement statistics.

**Complex**	**RPH-*****Lm*****·Mg**^2+^**·ADP**	**RPH-*****Lm*****·RIF**	**RPH-*****Lm*****·RIF-P**
**PDB Code**	**5FBS**	**5FBT**	**5FBU**
*Data collection*			
Space group	P4_1_2_1_2	P6_5_22	P6_5_22
Cell dimensions			
*a*, *b*, *c,* Å	58.5, 58.5, 601.9	149.3, 149.3, 193.5	151.6, 151.6, 191.5
Resolution, Å	50.54–2.59	25.00–2.70	49.63–2.85
*R*_sym_*	0.101 (0.717)†	0.063 (0.708)	0.078 (0.736)
*I*/σ(*I)*	12.3 (2.5)	24.8 (2.5)	19.0 (2.4)
Completeness, %	99.7 (100)	100 (100)	99.9 (99.9)
Redundancy	6.7 (7.2)	7.6 (6.9)	6.5 (6.7)
			
*Refinement*			
Resolution, Å	50.54–2.59	25.0–2.70	49.63–2.85
No. of unique reflections: working, test	34,390, 3,095	35,398, 3,262	30,933, 1,463
*R*-factor/free *R*-factor‡	26.1/31.9 (36.2/39.3)	22.0/26.0 (28.8/30.0)	23.3/29.3 (31.7/37.8)
No. of refined atoms, molecules			
Protein	6,161	5,801	5,442
Magnesium	1	N/A	N/A
Substrate	27	58	62
Other solvent	N/A	1	9
Water	49	177	167
*B*-factors			
Protein	77.4	69.3	76.3
Magnesium	35.9	N/A	N/A
Substrate	64.5	71.9	69.2
Other solvent	N/A	51.3	75.0
Water	51.0	53.2	59.1
r.m.s.d.			
Bond lengths, Å	0.006	0.006	0.008
Bond angles, °	1.167	1.161	1.417

RIF, rifampin; RIF phosphotransferase, RPH.

^*^R_sym_=Σ_h_Σ_i_|*I*_i_(*h*)−〈*I*(h)〉/Σ_h_Σ_i_I_i_(*h*), where *I*_i_(*h*) and 〈*I*(*h*)〉 are the *i*th and mean measurement of the intensity of reflection *h*.

^†^Figures in parentheses indicate the values for the outer shells of the data.

^‡^R=Σ|F_p_^obs^−F_p_^calc^|/ΣF_p_^obs^, where F_p_^obs^ and F_p_^calc^ are the observed and calculated structure factor amplitudes, respectively.

**Table 2:** RPH-*Lm* wild-type/mutant steady-state kinetics and RIF susceptibility in *Escherichia coli.*

**Enzyme**	**Implicated in ATP or RIF binding**	MIC μg RIF per ml*	**ATP** ***K***_**m**_ **(μM)**	**RIF** ***K***_**m**_ **(μM)**	**ATP** ***k***_**cat**_ **(s**^−1^**)**	**RIF** ***k***_**cat**_ **(s**^−1^**)**	***k***_**cat**_**/*****k***_**m**_ **ATP (s**^−1^ **M**^−1^**)**	***k***_**cat**_**/*****k***_**m**_ **RIF (s**^−1^ **M**^−1^**)**
No enzyme	NA	4	NA	NA	NA	NA	NA	NA
Wild-type RPH-Lm	NA	>512	8.38±0.67	0.57±0.15	2.87±0.04	2.25±0.17	3.42 × 10^5^	3.94 × 10^6^
Arg_117_Ala	ATP	4	56.12±8.36	NC	2.39±0.13	NC	4.3 × 10^4^	NC
Thr_136_Val	ATP	8	53.88±6.54	NC	2.1±0.1	NC	3.95 × 10^4^	NC
Lys_22_Ala	ATP	8	NC	NC	NC	NC	NC	NC
Glu_297_Ala	ATP	>512	49.49±6.85	4.76±1.26	43.21±2.16	45±4	8.73 × 10^5^	9.43 × 10^6^
Val_368_Thr	RIF	128	30.41±7.31	3.65±1.09	33.53±2.67	30.02±2.43	1.1 × 10^6^	8.22 × 10^6^
Val_368_Glu	RIF	256	32.79±5.41	4.56±1.78	39.9±2.1	45.72±4.85	1.21 × 10^6^	9.8 × 10^6^
Tyr_351_Phe	RIF	512	31.42±1.96	4.08±1.55	12.91±0.26	15.9±1.9	4.1 × 10^5^	3.89 × 10^6^
Arg_666_Ala	RIF	4	169.29±43.68	NC	2.71±0.37	NC	1.6 × 10^4^	NC
Glu_667_Ala	RIF	4	24.65±5.19	NC	1.78±0.12	NC	7.2 × 10^4^	NC
Gln_337_Ala	RIF	128	18.03±2.98	6±2	15.35±0.72	15.37±1.53	8.5 × 10^5^	2.7 × 10^6^

NA, not applicable; NC, not calculable; RIF, rifampin.

^*^Susceptibility of *E. coli* Rosetta(DE3) pLysS (pET19Tb:*rph-Lm*).

**Table 3:**
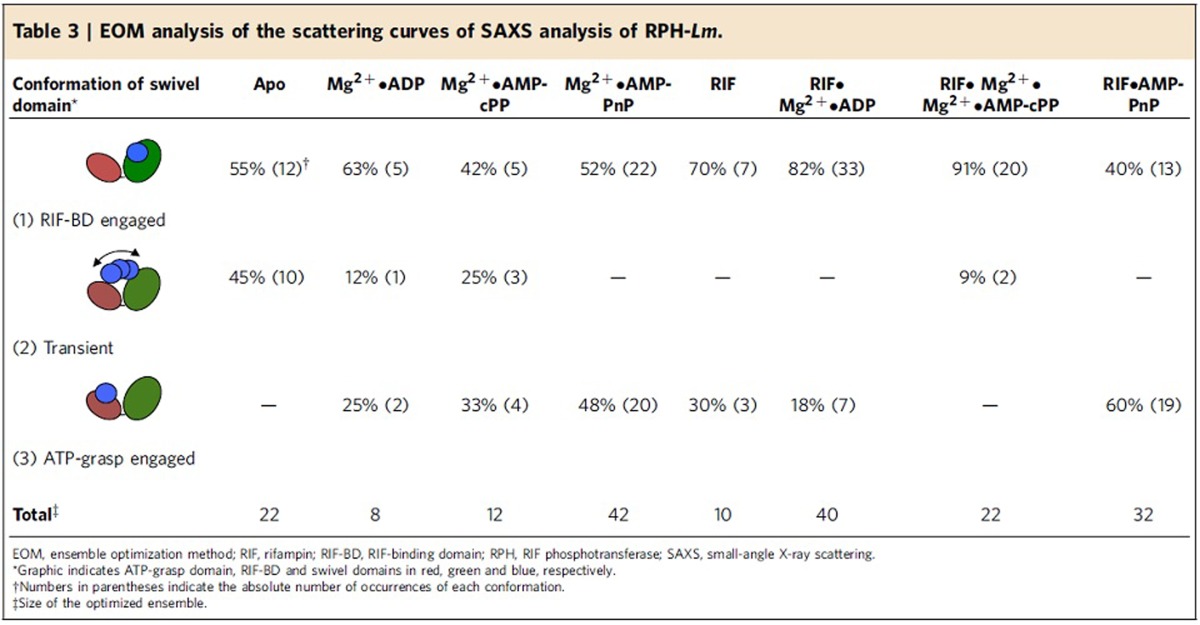
EOM analysis of the scattering curves of SAXS analysis of RPH-*Lm*.
