# Supplementary material for: Rifampin phosphotransferase is an unusual antibiotic resistance kinase
Source: Nat Commun. 2016 Apr 22;7:11343. doi: 10.1038/ncomms11343 (PMC4844700; doi:10.1038/ncomms11343)
Supplement: Supplementary Information — Supplementary Figure 1-4, Supplementary Tables 1-2 and Supplementary References. [file ncomms11343-s1.pdf]

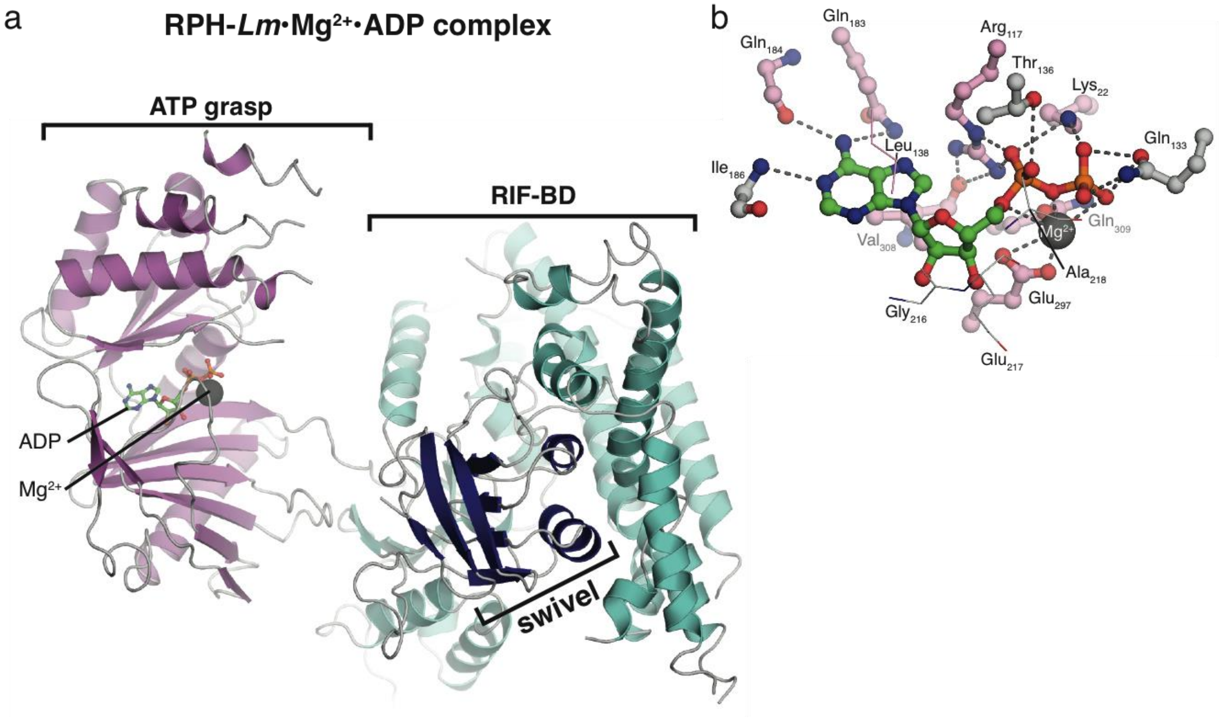

Supplementary Fig. 1 Structure of the RPH-*Lm*•Mg^{2+}•ADP complex. (**a**) ATP-grasp, RIF-binding domain (RIF-BD) and swivel phosphohistidine domains are shown in shades of purple, cyan and dark blue, respectively. ADP is shown in green sticks and Mg^{2+} ion as a grey sphere. (**b**) Binding site of Mg^{2+}•ADP in RPH-*Lm*.

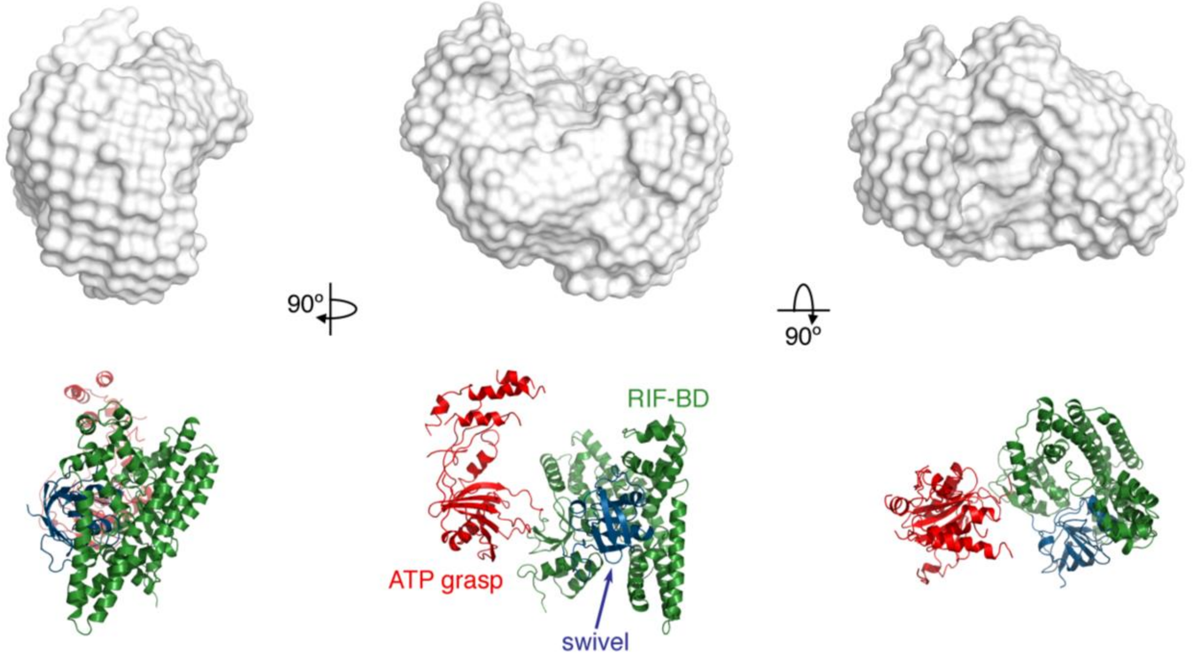

Supplementary Fig. 2 *Ab initio* model of apo-RPH**.** (**a**) Ten independent *ab initio* models of apo-RPH were generated using GASBOR[1] and clustered based on the normalized spatial frequency using DAMCLUST[2]. Orthogonal views of the most representative model for the dominant cluster are shown superimposed onto the crystal structure of RPH-*Lm*•RIF.

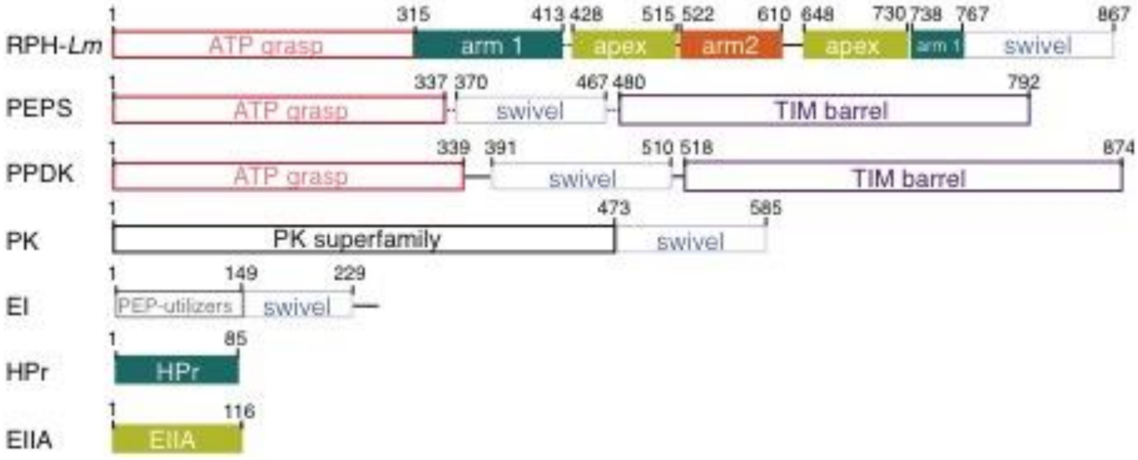

Supplementary Fig. 3 Primary sequence/domain architecture of RPH-*Lm* and structurally related proteins. Numbers indicate domain boundaries.

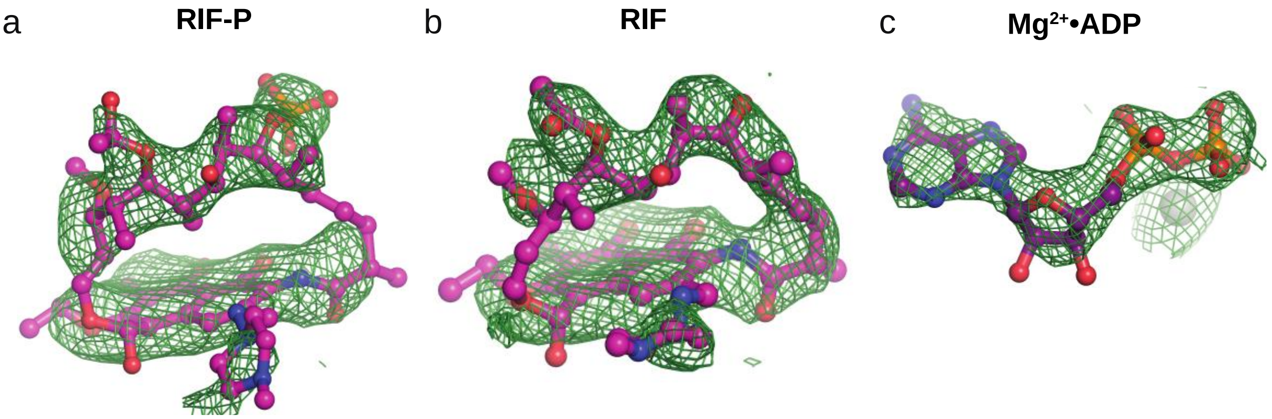

Supplementary Fig. 4 Electron density simulated annealing omit maps contoured at 2.0 σ of ligands in RPH-*Lm* crystal structures. (**a**) RIF-P from RPH-*Lm*•RIF-P complex. **(b)** RIF from RPH-*Lm*•RIF complex. (**c**) Mg^{2+}•ADP from RPH-*Lm*•Mg^{2+}•ADP complex.

Supplementary Table 1. LC/MS analysis of RIF-P formation catalyzed by RPH-*Lm* enzyme variants

RPH-*Lm* enzyme	% RIF-Phosphate present*
No enzyme	0
Wild-type	100
$Lys_{22}Ala$	0
$Arg_{117}Ala$	7
$Thr_{136}Ala$	1
$Glu_{297}Ala$	100
$Gln_{337}Ala$	100
$Tyr_{351}Phe$	100
$Val_{368}Thr$	100
$Val_{368}Glu$	100
$Arg_{666}Ala$	17
$Glu_{667}Ala$	14

*Extracted ion chromatogram at a retention time of 7.14 min and *m/z* ratio of 903.3 in positive mode. The % RIF-P present was based on the wild-type enzyme reactions being equal to 100% RIF-P formation and all enzyme variants were directly compared to this standard.

Supplementary Table 2. Solution scattering-derived parameters for RPH-*Lm*

					RIF			
	APO	ADP	AMPcPP	AMPPnP	APO	ADP	AMPcPP	AMPPnP
I_0 (cm^{-1}) [from Guinier]	0.6 ± 0.0	0.7 ± 0.0	0.2 ± 0.0	0.3 ± 0.0	0.4 ± 0.0	0.4 ± 0.0	0.4 ± 0.0	0.4 ± 0.0
R_g (Å) [from Guinier]	33.9 ± 0.5	32.7 ± 0.4	32.5 ± 0.5	32.7 ± 0.8	33.5 ± 1.3	33.8 ± 0.6	32.9 ± 0.9	32.7 ± 0.5
I_0 (cm^{-1}) [from P(r)]	0.6 ± 0.0	0.7 ± 0.0	0.2 ± 0.0	0.3 ± 0.0	0.4 ± 0.0	0.4 ± 0.0	0.4 ± 0.0	0.4 ± 0.0
R_g (Å) [from P(r)]	33.5 ± 0.5	32.7 ± 0.1	32.9 ± 0.0	32.5 ± 0.1	33.2 ± 0.1	33.1 ± 0.1	32.8 ± 0.2	32.6 ± 0.1
D_{max} (Å)	95	93	94	95	102	104	104	99
Experimental MW [from Q_R]*	110 kDa	108 kDa	103 kDa	95 kDa	98 kDa	101 kDa	95 kDa	101 kDa
χ^2 of EOM analysis	1.1	1.3	1.0	1.1	1.1	1.0	1.0	1.3

*Calculated MW is 96.9 kDa

Supplementary References

1. Svergun, D.I., Petoukhov, M.V. & Koch, M.H. Determination of domain structure of proteins from X-ray solution scattering. *Biophys J* **80**, 2946-53 (2001).
2. Petoukhov, M.V. et al. New developments in the program package for small-angle scattering data analysis. *J Appl Crystallogr* **45**, 342-350 (2012).
